# Nutrition phenotypes in inflammatory bowel disease: emerging concepts and future directions to guide clinical practice

**DOI:** 10.3389/fnut.2026.1895716

**Published:** 2026-08-10

**Authors:** Stephanie Gold, Natasha Haskey, Hannah Freid, Max Eisele, Kristen Nicolai, David Kohler, Maitreyi Raman

**Affiliations:** 1 The Henry D. Janowitz Division of Gastroenterology, Icahn School of Medicine at Mount Sinai, New York, NY, United States; 2 Department of Biology, Irving K. Barber Faculty of Sciences, University of British Columbia Okanagan, Kelowna, BC, Canada; 3 Department of Internal Medicine, Icahn School of Medicine at Mount Sinai, New York, NY, United States; 4 Cumming School of Medicine, University of Calgary, Calgary, AB, Canada; 5 Department of Nutrition, Mount Sinai Hospital, New York, NY, United States; 6 Department of Pediatrics, Icahn School of Medicine at Mount Sinai, New York, NY, United States

**Keywords:** nutrition phenotype, nutrition therapy, Inflammatory Bowel Disease, Crohn’s disease, ulcerative colitis, nutrition, malnutrition

## Abstract

Nutrition occupies a central and dynamic role across the inflammatory bowel disease (IBD) care continuum, from disease risk reduction and prevention, through induction and maintenance of remission, to the mitigation of disease-related and unrelated complications and the preservation of quality of life – yet translating this evidence into individualized clinical practice remains a persistent challenge. To address this gap, we conducted a narrative review of the current literature and propose the concept of the nutrition phenotype: a structured framework that integrates disease-specific characteristics, nutritional status, body composition, dietary patterns, and lifestyle factors to define clinically distinct patient profiles. Applying this framework to six phenotypes: malnutrition, overnutrition, dietary therapeutics, functional symptoms, high enteric loss, and complication prevention, we synthesize the evidence supporting targeted nutritional interventions for each and present an integrated clinical algorithm plus a proposed hierarchy for managing patients with overlapping phenotypes to guide prioritization and decision-making. This phenotype-driven approach provides a practical, personalized structure for nutrition care in inflammatory bowel disease, with the aim of improving implementation feasibility and patient outcomes across real-world clinical settings.

## 1 Introduction

Diet is recognized as both a modifiable environmental determinant of disease risk and a therapeutic tool in inflammatory bowel disease (IBD). This recognition has transformed clinical practice: recent guidelines from major gastroenterology societies have elevated dietary approaches to a position alongside pharmacologic and surgical approaches as integral components of IBD management (1–3). The therapeutic landscape spans a continuum of nutrition interventions for induction and maintenance of remission from formula-based regimens, such as exclusive enteral nutrition (EEN) and partial enteral nutrition (PEN), to whole-food diet approaches like the Crohn’s Disease Exclusion Diet (CDED), Mediterranean diet (MD), and the Tasty and Healthy™ (T&H™) diet, each with distinct mechanisms, targets and evidence bases (1, 4). EEN, for example, achieves remission rates comparable to corticosteroids in pediatric Crohn’s disease (CD), likely through modulation of the intestinal microenvironment, mucosal immune function, and luminal antigenic load; yet issues of palatability, tolerability, and inter-individual variability in response continue to limit its real-world uptake (5, 6).

Compounding the therapeutic challenge is the nutritional complexity inherent to IBD itself. Malnutrition affects an estimated 20%–70% of patients, while more than 40% of patients are overweight or obese, two ends of a nutritional spectrum that carry distinct but equally significant risks for adverse disease and clinical outcomes (7, 8). The broad range in reported malnutrition prevalence likely reflects the heterogeneity of the included patient populations, including differences in disease phenotype, disease type, severity, and clinical characteristics and the variable tools used to assess malnutrition; overall, the studies report roughly 70%–80% of hospitalized IBD patients have concomitant malnutrition while roughly 30% of outpatients with malnutrition (9). Up to one-third of patients experience symptoms consistent with concurrent irritable bowel syndrome (IBS), introducing a functional layer that is often refractory to anti-inflammatory therapy alone (10). Meanwhile, 50%–80% of patients with CD will require small bowel resection at some point in their disease course, introducing anatomical and absorptive consequences, including risk of high ileostomy output and/or short bowel syndrome that fundamentally reshape nutritional requirements and dietary tolerance (11). At any given time, a single patient may simultaneously contend with multiple, and sometimes competing, nutritional challenges, underscoring the inadequacy of a one-size-fits-all dietary approach. These findings highlight the urgent need for a structured, individualized framework for the assessment and management of nutrition in IBD.

**FIGURE 1:**
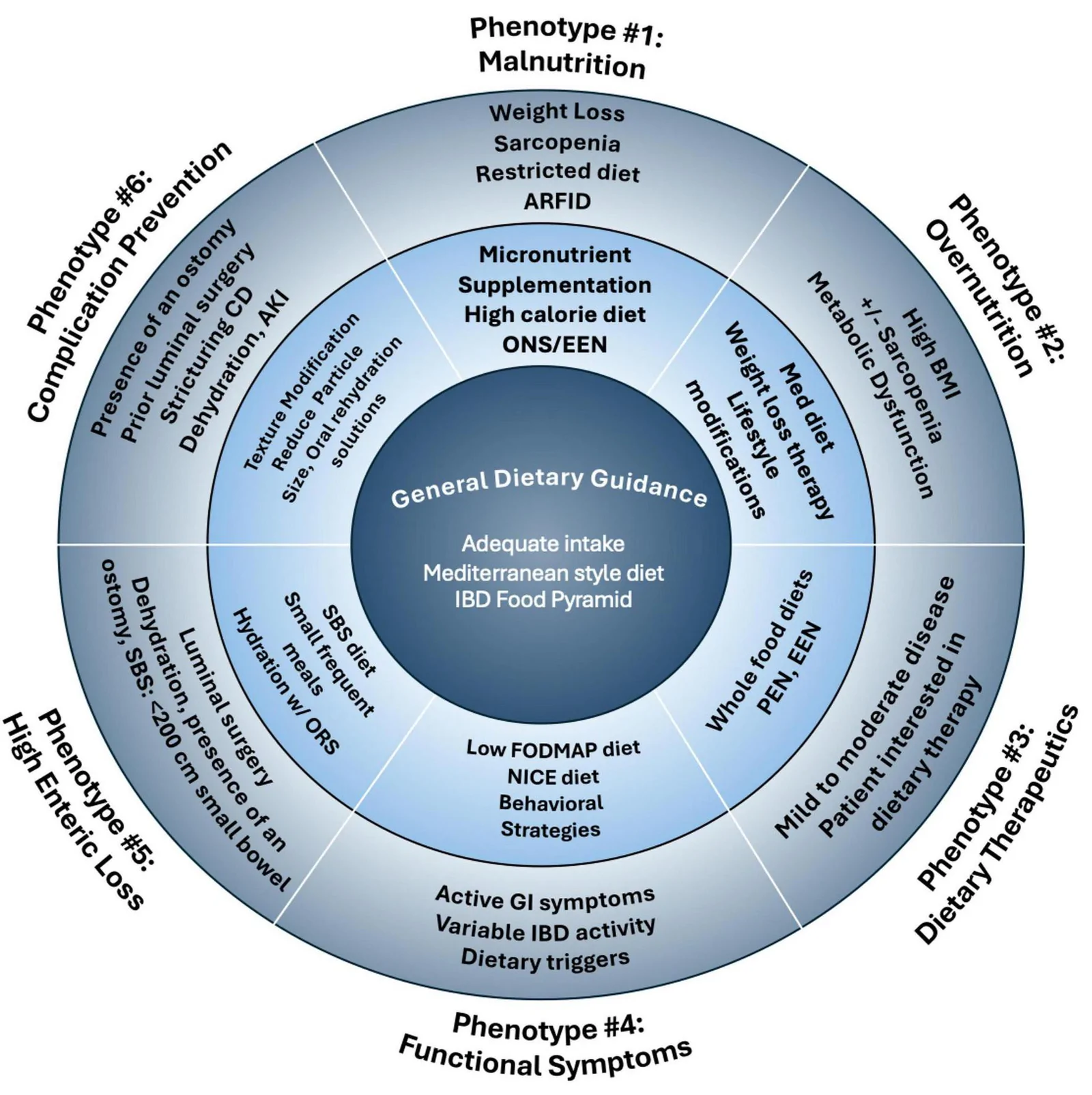
Nutrition phenotypes for inflammatory bowel disease. Schematic representation developed as part of this narrative review illustrating proposed nutrition phenotypes in pediatric and adult patients with inflammatory bowel disease. The outer core depicts principal nutrition phenotypes, with the subsequent ring outlining associated risk factors, clinical profiles, and nutritional status. Surrounding concentric rings represent progressive nutrition interventions, including Mediterranean-style dietary patterns, ONS^a^, EEN^b^, high-calorie diets, and targeted micronutrient supplementation. The model underscores that nutrition care in inflammatory bowel disease is dynamic and cyclical, emphasizing personalized, layered strategies to optimize digestive health, nutritional status, and support remission induction and maintenance. *^a^*ONS, oral nutrition supplements; *^b^*EEN, exclusive enteral nutrition.

To address this need, we propose the concept of the “IBD nutrition phenotype” (Figure 1), which provides a clinically grounded framework for categorizing patients according to their dominant nutritional profile and tailoring dietary interventions accordingly. Nutrition phenotypes are defined across four intersecting domains: dietary patterns (encompassing whole-food quality, degree of processing, and macro- and micronutrient composition); host metabolic and nutritional status (including malnutrition, micronutrient deficiencies, sarcopenia, and obesity); anatomy-guided nutritional complications (such as high ostomy output and intestinal strictures); and symptom-driven manifestations of gut–brain interaction. The integration of these domains generates distinct nutritional profiles that capture the variation in physiological state, dietary requirements, and intervention priorities across the IBD population. Importantly, this phenotype-driven approach does not impose a rigid classification but rather provides a flexible, physiologically informed framework that accommodates the dynamic and often overlapping nature of nutritional challenges in IBD.

The clinical utility of this framework is illustrated by the divergence in optimal dietary strategy across phenotypes. Patients in biochemical remission but burdened by persistent IBS-like symptoms represent a distinct functional phenotype best served by low Fermentable Oligosaccharides, Disaccharides, Monosaccharides, and Polyols (FODMAP) or FODMAP gentle approach, while those with active luminal inflammation may derive benefit from immune-modulatory dietary approaches such as CDED + PEN or T&H™, both of which have demonstrated reductions in biomarkers of intestinal inflammation (4, 12, 13).

The objective of this narrative review is to synthesize current evidence on the nutritional management of IBD, identifying six potential nutritional phenotypes. For each phenotype, the clinical and nutritional rationale, supporting evidence and priority interventions are addressed. We conclude by presenting an integrated clinical algorithm for this hypothesis-generating model of nutrition care, highlighting emerging opportunities, including precision nutrition and multidisciplinary care models, to advance the field toward more individualized, outcomes-driven dietary practice in IBD.

## 2 Methods

A literature review was performed from inception to May 1, 2026, using OVID Medline, Embase, and Scopus databases. Original research studies (both observational and intervention studies) and review articles in English involving human participants were included. The search headings “inflammatory bowel disease,” OR “Crohn’s disease,” OR “ulcerative colitis” with a combination of: “dietary therapy,” OR “nutrition” OR “nutrition therapeutics” OR “obesity” OR “metabolic disease” OR “malnutrition” OR “irritable bowel syndrome” OR “ileal pouch anal anastomosis” OR “dehydration” OR “ileostomy” were included. References included in the identified publications were manually reviewed and any relevant publications were included. Randomized clinical trials, prospective studies, cross-sectional studies, and retrospective studies were prioritized for inclusion, while case series and case reports were excluded.

## 3 Nutrition phenotypes in IBD: linking nutrition phenotypes to dietary interventions

The nutritional landscape of IBD is both heterogeneous and dynamic, shaped by disease activity, anatomy, body composition, and symptom burden. Each nutrition phenotype is associated with unique dietary priorities and, in certain instances, specific contraindications in which standard approaches may be unsuitable or require thoughtful modification. The six phenotypes are summarized and visually represented in Figure 1.

To operationalize phenotype prioritization in clinical practice, a suggested hierarchy is proposed: (1) correct acute physiological instability first, dehydration, severe malnutrition, and electrolyte disturbance as these take precedence over all other phenotype-directed interventions; (2) address active luminal inflammation next, as uncontrolled disease drives ongoing nutritional deterioration across phenotypes; (3) then layer phenotype-specific dietary guidance according to the patient’s dominant nutritional profile, reassessing at each clinical encounter. Figure 1 serves as the integrated clinical algorithm providing a visual representation of the six phenotypes, their associated risk factors, and the progressive nutritional interventions that map to each. Suggested criteria, recommended first-line assessment tools, and indicative thresholds for assigning each phenotype are summarized in Table 1.

**TABLE 1:** Suggested criteria, recommended first-line assessment tools, and indicative thresholds for identifying nutrition phenotypes in inflammatory bowel disease.

Nutrition phenotype	Defining criteria	First-line assessment tool(s)	Indicative thresholds/cut-offs
1. Malnutrition	Reduced intake, malabsorption, increased nutritional requirements, with weight loss, low BMI, or reduced muscle mass; includes sarcopenia and micronutrient deficiencies	Screen with MUST or MST; diagnose with SGA, RD comprehensive assesment, GLIM, body comp (BIA, DXA). Handgrip dynamometry can be used with other measures. Serum nutrient levels.	MUST ≥ 2 = high risk. GLIM: unintentional weight loss > 5% in 6 months (or >10% beyond 6 months), BMI < 20 kg/m^2^ (<70 y) or <22 (≥70 y), plus reduced muscle mass
2. Overnutrition	Overweight or obesity with excess visceral adipose tissue (VAT) and metabolic dysfunction (±MASLD)	BMI and waist circumference; cross-sectional imaging (CT/MRI) or DXA to quantify VAT	Caucasians: BMI ≥ 25 kg/m^2^ (overweight) or ≥30 (obesity); Asians: BMI ≥ 23 kg/m^2^ (overweight) or ≥27 (obesity); elevated VAT on imaging
3. Dietary therapeutics	Active luminal inflammation in a patient who is a candidate for diet as primary or adjunctive anti-inflammatory therapy	Fecal calprotectin, CRP; endoscopic, cross-sectional imaging	Elevated fecal calprotectin (e.g., >150–250 μg/g) and/or CRP (>8) endoscopic disease activity
4. Functional symptoms (DGBI)	Persistent GI symptoms despite biochemical and endoscopic remission (IBS-type overlap)	Rome IV criteria; confirm remission with fecal calprotectin, cross-sectional imaging ± endoscopy before dietary restriction; absence of malnutrition	Rome IV criteria; fecal calprotectin in remission range (e.g., <150–250 μg/g) confirmed before initiating a low-FODMAP diet
5. High output/enteric loss	High-output ileostomy, ileal pouch–anal anastomosis, or short bowel syndrome	Measure stoma/ostomy output and urine output; serum electrolytes; document residual small-bowel length	Stoma output > 1.4 L/day; increased bowel frequency/watery consistency, small bowel < 200 cm
6. Complication prevention	Stricturing disease, dehydration risk, reduced bone mineral density	Cross-sectional imaging or endoscopy for strictures; electrolyte and hydration monitoring; DXA for bone density	Symptomatic luminal or anastomotic stricture; DXA T-score ≤ −2.5 (osteoporosis) or −1 to −2.5 (osteopenia)

Thresholds are illustrative and intended to support, not replace, individualized clinical judgment. BIA, bioelectrical impedance analysis; BMI, body mass index; CRP, C-reactive protein; CT, computed tomography; DGBI, disorders of gut–brain interaction; DXA, dual-energy X-ray absorptiometry; FODMAP, fermentable oligosaccharides, disaccharides, monosaccharides, and polyols; GLIM, Global Leadership Initiative on Malnutrition; IBS, irritable bowel syndrome; MASLD, metabolic dysfunction-associated steatotic liver disease; MRI, magnetic resonance imaging; MUST, Malnutrition Universal Screening Tool; MST, Malnutrition Screening Tool; SGA, Subjective Global Assessment; VAT, visceral adipose tissue.

### 3.1 Phenotype 1: malnutrition

Malnutrition is one of the most common and clinically significant complications of IBD, with reported estimates ranging from 20% to 70% depending on disease activity, phenotype, and disease complications (14). Its etiology is multifactorial, arising from the convergence of reduced oral intake as a result of pain, fear of eating, symptom-provoked food aversion, self-imposed elimination diets, along with chronic enteric losses, malabsorption, altered luminal anatomy after surgery, and increased catabolic needs (hypermetabolism) in the setting of systemic inflammation (15). The clinical burden of malnutrition extends well beyond nutritional depletion: it has been independently associated with impaired immune function, increased rates of disease recurrence, postoperative complications, sarcopenia, prolonged hospitalization, readmission, and diminished quality of life (16, 17).

In recognition of this burden, current nutrition and IBD guidelines recommend screening for malnutrition at diagnosis and at regular intervals throughout the disease course. Accurate identification requires systematic, universal screening and assessment tools, such as the Malnutrition Universal Screening Tool (MUST) and Subjective Global Assessment (SGA) (18). The Global Leadership Initiative on Malnutrition (GLIM) criteria, which integrate objective phenotypic measures (BMI, weight loss, reduced muscle mass) with etiologic factors (reduced intake, decreased absorption, and inflammation), have been proposed as a standardized diagnostic framework and are increasingly being applied and validated in IBD cohorts (18, 19). The growing recognition of sarcopenia has driven interest in practical, point-of-care tools for assessing muscle mass and function, including bioelectrical impedance analysis, handgrip dynamometry, dual-energy X-ray absorptiometry (DXA), and muscle ultrasound (20).

Nutritional treatment must be individualized to address both the underlying inflammatory state and the patient’s specific pattern of deficits. During active disease or in the presence of established malnutrition, energy and protein requirements are substantially elevated: international guidelines, including the 2023 ESPEN consensus, recommend an energy target of approximately 30 kcal/kg/day alongside high-quality protein intake of 1.2–1.5 g/kg/day, with oral nutrition supplements (ONS) typically required to achieve these targets in clinical practice (20). Micronutrient deficiencies are near-universal in active IBD and arise from the compounding effects of intestinal inflammation, malabsorption, dietary restriction, and prior bowel resection. Iron, vitamin B12, folate, vitamin D, zinc, phosphate, and magnesium represent the most frequently depleted nutrients and should be systematically screened for and corrected. For patients with sarcopenia or low lean mass, optimization of disease control must accompany nutritional rehabilitation; targeted protein intake of at least 1.2 g/kg/day, in conjunction with structured resistance exercise, is recommended to support muscle protein synthesis and functional recovery. Parenteral nutrition remains indicated in the uncommon scenario in which the enteral route is contraindicated, such as bowel obstruction or a high-output proximal fistula, though enteral feeding is always preferred for its established benefits to gut mucosal integrity, immune homeostasis, and infectious risk (20). Early, proactive nutritional intervention not only corrects deficits but demonstrably reduces hospital length of stay, lowers postoperative morbidity, and improves quality of life; it should therefore be prioritized whenever this phenotype is identified, including when it coexists with competing phenotypes (20).

### 3.2 Phenotype 2: overnutrition

Beyond general nutritional status, body composition and increased visceral adipose tissue (VAT) have emerged as clinically meaningful modifiers of IBD disease course and treatment response. A growing body of evidence implicates excess VAT as an active immunoendocrine compartment that sustains a pro-inflammatory milieu through dysregulated secretion of adipokines, cytokines, and chemokines, creating pathophysiological overlap between metabolic dysfunction and intestinal inflammation (7, 21). The Constellation Study, a prospective cohort enrolling 141 patients with IBD initiating infliximab, vedolizumab, or ustekinumab, demonstrated that higher intra-abdominal VAT percentage was independently associated with lower rates of corticosteroid-free deep remission and endoscopic remission across all three biologic classes, an effect not explained by differences in pharmacokinetics (21). Observational data further link obesity with higher rates of surgical complications, increased hospitalization, greater symptom burden, and reduced quality of life, underscoring the need to address metabolic phenotype as an integral component of IBD management (22). Furthermore, metabolic-associated steatotic liver disease (MASLD) is now recognized as the most common extraintestinal hepatic manifestation of IBD, with prevalence estimates that continue to rise in parallel with the increasing burden of metabolic comorbidity in this population (23, 24) Nutritional strategies targeting VAT reduction, aligned with the overnutrition phenotype, and consistent adherence to Mediterranean-style dietary principles represent the cornerstones of MASLD prevention and management in IBD.

Lifestyle interventions targeting VAT, including Mediterranean-style dietary patterns and structured physical activity programs, have shown modest but meaningful improvements in waist circumference, BMI, and inflammatory biomarkers in small IBD cohorts, though high-quality interventional data specifically powered for VAT reduction in this population remain limited (23). Popular weight loss strategies have been extrapolated from the general population, including the Paleo, Atkins, and ketogenic diets, have not been systematically evaluated in IBD. The most promising emerging evidence concerns intermittent fasting (IF): a randomized controlled study of time-restricted eating in adults with mild-to-moderate CD demonstrated significant reductions in BMI and VAT alongside favorable modulation of pro-inflammatory cytokines (IL-2, IL-12p40, IL-12p70) and adipokines (leptin, adipsin, and plasminogen-activator inhibitor-1), providing preliminary evidence that dietary timing strategies may target adipose-driven inflammation through mechanisms distinct from caloric restriction alone (25, 26). Clinicians should therefore apply this guidance with appropriate caution, individualizing recommendations in light of each patient’s disease activity, medication burden, and nutritional risk, and recognizing that strategies well-tolerated in the general population may require modification in the context of active or complicated IBD. The increasing interest and preliminary nature of the evidence on glucagon-like peptide-1 (GLP-1) receptor agonists as both metabolic and potentially immunomodulatory agents in IBD represents a compelling area for future study.

### 3.3 Phenotype 3: dietary therapeutics (induction or maintenance of remission)

Dietary therapies for IBD span a continuum from formula-based regimens to whole-food approaches, with an expanding evidence base supporting their role in both the induction and maintenance of remission. To date, there are numerous studies assessing the evidence for use of various dietary strategies for induction and maintenance of remission in IBD; while this review touches on a few dietary interventions as examples for this phenotype, the breadth and depth of the evidence is best reviewed in the current published literature (27–30).

What distinguishes Phenotype 3 from the other five phenotypes is that it applies specifically to patients with active luminal inflammation who are candidates for diet as a primary or adjunctive anti-inflammatory therapy, that is, diet used not merely to optimize nutritional status or relieve symptoms, but to directly modulate intestinal inflammation and induce or sustain mucosal remission. The interventions characteristic of this phenotype (e.g., EEN, CDED, T&H™) exert their effects through modulation of the intestinal microenvironment, luminal antigenic load, and mucosal immune function, and are distinct in both mechanism and intent from dietary strategies deployed across other phenotypes.

Exclusive enteral nutrition remains the most rigorously evidenced dietary intervention for remission induction in pediatric CD, achieving remission rates comparable to corticosteroids while additionally promoting mucosal healing (31, 32). In adults, EEN efficacy is recognized but real-world uptake is substantially constrained by palatability, tolerability, and cost. PEN combined with whole-food dietary strategies, most notably the CDED addresses these barriers and has demonstrated sustained remission in both pediatric and adult populations with mild-to-moderate CD (33, 34). In an innovative extension of the EEN paradigm, a whole-food smoothie reverse-engineered to replicate the macronutrient composition of EEN achieved comparable clinical and biochemical responses, including reductions in fecal calprotectin in a pediatric CD pilot, suggesting that formula-free approaches to mimicking enteral nutrition may broaden accessibility (35). Beyond its role in remission induction, EEN remains a cornerstone therapeutic in malnourished patients with moderate-to-severe or fistulizing CD, where bowel rest, optimized nutrient delivery, and correction of micronutrient deficits serve as stabilizing bridges prior to pharmacological escalation or surgical intervention.

Emerging evidence points to synergistic benefits when PEN is combined with advanced pharmacologic therapies, including biologics and small-molecule agents, with combination approaches associated with higher rates of clinical and biochemical remission than pharmacotherapy alone (36). A pilot study in patients with refractory CD who had lost response to biologic therapy demonstrated that addition of PEN during dose escalation was associated with both clinical and transmural remission alongside improvements in nutritional status, findings that illustrate the complementary mechanisms through which nutritional therapy and targeted immune modulation act together to restore mucosal integrity (36).

Among the whole-food diets, the T&H™ diet, which excludes processed foods, animal fats, and gluten, while emphasizing nutrient-dense foods such as vegetables, fruits, legumes, rice, eggs, poultry, seafood, and fish, demonstrated superior tolerability compared to EEN and was effective in inducing remission in mild-to-moderate CD (4). The CD-TREAT diet, designed to mimic EEN using ordinary foods, produced comparable reductions in CRP and fecal calprotectin (FCP) in a small pediatric cohort with CD, further establishing proof of concept for formula-free, composition-matched diet therapy (37). Other elimination-based diet approaches, including the autoimmune protocol (AIP), the IBD Anti-Inflammatory Diet (IBD-AID), and the specific carbohydrate diet (SCD), have shown improvements in patient-reported symptoms; however, biochemical, endoscopic, or histologic evidence remains limited and robust comparative trial data are lacking (37–40).

For maintenance of remission, broad dietary guidance is informed by large epidemiologic and interventional studies implicating ultra-processed food consumption as a driver of IBD risk and relapse.

The International Organization for the Study of IBD (IOIBD) recommends limiting emulsifiers, thickeners, trans fats, processed foods, and unpasteurized dairy, while emphasizing nutrient-dense whole foods (41). The MD and the IBD Food Pyramid operationalize these principles in a practical, sustainable dietary pattern (1, 42). In both ulcerative colitis (UC) and CD, adherence to an MD diet is associated with reduced fecal calprotectin and beneficial modulation of the gut microbiome (43–45). Although the MD was not found to be superior to the SCD in a head-to-head randomized trial in mild-to-moderate CD, adherence to the MD was associated with reduced inflammatory markers, beneficial microbial and metabolic shifts, and improvements in cardiometabolic health, outcomes of independent significance in an IBD population at elevated cardiovascular risk (46). Emerging data for UC suggest that low-sulfur dietary patterns, which reduce intake of sulfur-rich animal proteins and food additives such as sulfites and carrageenan, may attenuate microbial hydrogen sulfide production and strengthen epithelial barrier integrity, identifying dietary composition as a modulator of the mucosal microenvironment beyond caloric or macronutrient content alone (47, 48).

Despite the expanding evidence base, the field is constrained by methodological limitations, as most available studies are small, heterogeneous, and lack standardized endpoints and comparator groups (38). No single diet has demonstrated universal efficacy, highlighting the need for individualized, patient-centered approaches. Precision nutrition approaches that integrate host genomics, metagenomic profiling of the microbiome, and immune phenotyping, offer a scientifically compelling framework for matching dietary interventions to individual biological signatures and represent the horizon toward which the field is moving (49).

### 3.4 Phenotype 4: functional symptoms

Disorders of gut–brain interaction (DGBIs), the preferred term replacing functional gastrointestinal disorders (FGIDs), comprise a heterogeneous spectrum of conditions characterized by disturbances in motility, visceral hypersensitivity, mucosal and immune function, luminal microbiota, and central nervous system processing (50). Their co-occurrence with IBD is clinically significant and often underappreciated; up to one-quarter of patients with quiescent IBD experience persistent gastrointestinal symptoms attributable not to active mucosal inflammation but to concurrent DGBI (51). This distinction is clinically critical as it determines whether escalation of anti-inflammatory therapy is warranted or whether symptom-directed dietary and behavioral strategies represent the appropriate therapeutic priority.

IBS is the most prevalent DGBI, and its overlap with IBD represents a particularly common and challenging clinical scenario. A 2020 meta-analysis reported IBS-like symptoms in 35% of patients in clinical remission and 25% in endoscopic remission, with a higher prevalence in CD than UC (10). The low FODMAP diet remains the most evidence-based dietary intervention for this phenotype (once there is confirmation of biochemical or endoscopic IBD remission), with demonstrated efficacy in reducing functional GI symptom burden in quiescent IBD (52–54). Its structured implementation, comprising an initial 2–6 weeks restriction phase followed by systematic reintroduction to establish an individualized tolerance threshold, is central to its effectiveness and safety (53). Critically, the immunological neutrality of this approach must be communicated clearly to patients and clinicians: the low FODMAP diet targets symptom burden and does not confer anti-inflammatory benefit, does not reduce mucosal inflammation, and, when prolonged, may reduce microbial diversity and short-chain fatty acid production, reinforcing the necessity of dietitian-led supervision to safeguard nutritional adequacy and mitigate the risk of avoidant restrictive food intake disorder (ARFID) (54). A 2025 mixed-methods systematic review and meta-analysis further corroborated the symptomatic benefit of the low FODMAP diet in IBD while noting heterogeneity in study populations and outcome measures, underscoring the continued need for IBD-specific trial data (55). Less restrictive alternatives, including the gentle FODMAP approach and NICE guideline-based dietary strategies, represent pragmatic options for patients in whom full FODMAP restriction is not feasible or is poorly tolerated, and may offer equivalent symptomatic benefit for selected individuals.

### 3.5 Phenotype 5: high output/enteric loss

#### 3.5.1 High-output ileostomy

Patients with IBD who develop a high-output ileostomy, defined as stoma output exceeding 1.4 L/day, face significant nutritional challenges due to excessive fluid and nutrient losses (56). Because the magnitude of nutrient loss is proportional to the degree of malabsorption, total energy requirements may exceed standard predictions by 20%–50%, and protein intake targets of 1.2–1.5 g/kg/day are typically necessary to preserve lean mass and support mucosal recovery. Fluid management is simultaneously critical: both hypotonic fluids (including water and dilute beverages) and hypertonic drinks (juices, sodas, and sugar-sweetened beverages) paradoxically amplify stomal output through osmotically driven fluid shifts and should be limited. Sodium-glucose oral rehydration solutions, formulated to exploit intestinal co-transport mechanisms, represent the preferred vehicle for hydration in this population. Patients benefit from small, frequent meals with large volumes of liquids separated from solids to slow transit time. Complex carbohydrates and soluble fiber are preferred to improve effluent consistency, while insoluble fiber should be minimized during high-output states. Regular monitoring of electrolytes and micronutrients, particularly sodium, potassium, magnesium, zinc, and selenium, is essential.

#### 3.5.2 Ileal pouch–anal anastomosis

Proctocolectomy with ileal pouch–anal anastomosis substantially alters gastrointestinal physiology, and nutrition plays an integral role in optimizing pouch function and quality of life following surgery (57). Common post-operative challenges include increased stool frequency, urgency, and altered consistency. Dietary strategies focused on reducing insoluble fiber, limiting fat and alcohol intake, and adjusting meal frequency and size can meaningfully attenuate these symptoms while preserving nutritional adequacy (58). Individualized assessment of food tolerance, which varies substantially between patients, is essential; a symptom-guided approach, ideally supported by a dietitian experienced in pouch care, is preferable to population-level dietary restrictions.

#### 3.5.3 Short bowel syndrome

Short bowel syndrome (SBS), defined as residual small intestinal length of 200 cm or less, represents the most physiologically complex nutritional phenotype in IBD. Management focuses on optimizing energy and protein intake and maintaining fluid and electrolyte balance (59). Patients with preserved colon continuity are best served by diets rich in complex carbohydrates and soluble fiber, which enhance colonic salvage of unabsorbed carbohydrate energy through fermentation and facilitate fluid and sodium absorption. In contrast, patients without a functional colon may require modified fat intake in selected cases, with medium-chain triglycerides (MCTs) offering an absorption advantage by bypassing the conventional pathway for long-chain fat digestion (60). Practical dietary principles consistently recommended across SBS subtypes include small, frequent meals (5–6 per day), separation of liquids from solids, and use of sodium-glucose oral rehydration solutions in preference to plain water (60). Systematic micronutrient monitoring and supplementation, encompassing vitamin B12, fat-soluble vitamins, folate, iron, zinc, copper, selenium, and magnesium, is essential given the high and ongoing risk of deficiency. Adjunctive medical therapies such as anti-motility agents and, in selected cases, teduglutide (a glucagon-like peptide-2 analog) can further enhance absorption. Given the complexity of management, a multidisciplinary intestinal rehabilitation team is strongly recommended (60).

### 3.6 Phenotype 6: complication prevention

Stricture formation in CD, a consequence of transmural fibrosis, affects an estimated 35% of patients within 10 years of diagnosis and carries significant nutritional implications that are frequently under addressed in clinical practice (61). Patients with symptomatic luminal or anastomotic strictures are often unable to tolerate the coarse texture of fibrous plant foods, placing them at risk for progressive dietary restriction and nutritional deterioration. Rather than defaulting to broad food exclusion, emphasizing careful chewing, thorough cooking (fork-tender), and mechanical modification (e.g., blending, mashing, or pureeing) of vegetables can improve tolerance and allow patients to maintain a more diverse, fiber-inclusive diet (1). More specifically, patients should be instructed to: (a) peel and cook all vegetables until fork tender and consider blending into a soup; (b) peel all fruit, which should be ripe and soft, or blend into a smoothie; (c) avoid whole nuts and instead use smooth/creamy nut butters; (d) choose ground or slow cooked proteins and (e) choose softer breads without nuts/seeds or a dense crust (62, 63).

Prevention of dehydration and its sequelae, including acute kidney injury (AKI) and unplanned healthcare utilization for electrolyte resuscitation, is a shared priority across multiple IBD phenotypes but is particularly critical for patients with post-surgical anatomy. Sodium-glucose oral rehydration solutions remain the preferred approach for maintaining hydration while avoiding the osmotic complications associated with both plain water and high-osmolarity beverages (64). Finally, reduced bone mineral density is a frequent extraintestinal complication of IBD. While prevalence estimates vary by cohort, osteopenia affects approximately 22%–77% of patients and osteoporosis 17%–41%; a recent systematic review and meta-analysis reported pooled prevalences of 31.5% and 12.2%, respectively. These findings reflect the combined impact of chronic corticosteroid use, dietary restriction, malabsorption, and ongoing systemic inflammation on skeletal health (65). Dietary counseling should proactively address calcium and vitamin D adequacy, and weight-bearing exercise with progressive resistance training should be recommended as complementary strategies to support bone mineral density and reduce fracture risk across the IBD population.

## 4 Discussion

Nutrition is no longer a supportive adjunct in IBD management: it is an active therapeutic domain, integral to disease control, complication prevention, and the preservation of quality of life across the disease continuum. This narrative review synthesizes current evidence on dietary and nutritional management in IBD through the novel lens of nutrition phenotypes, a structure designed to move clinical practice beyond generalized dietary advice toward interventions that are calibrated to each patient’s metabolic state, anatomical context, symptom profile, and dietary capacity.

The six nutrition phenotypes delineated in this review capture the heterogeneity of IBD and the need for individualized approaches. For example, those with active inflammation and malnutrition benefit from high-energy, high-protein approaches, often including EEN or PEN, to support mucosal healing and nutritional recovery. Similarly, patients with increased VAT and metabolic dysfunction may benefit from Mediterranean-style diets and time-restricted eating to reduce VAT-driven inflammation and improve cardiometabolic health. Anatomically driven phenotypes, such as high-output ileostomy or short bowel syndrome, require a focus on hydration, electrolyte balance, and nutrient absorption, while functional symptom phenotypes are best managed with symptom-directed strategies such as a dietitian-supervised low FODMAP diet.

The nutrition phenotype framework offers a unifying, physiology-based approach that integrates diverse nutritional challenges under a single conceptual model. It enables clinicians to tailor interventions to individual needs while providing a structure for research that reduces patient heterogeneity in dietary trials. Integration of IBD-competent dietitians into patient-centric, multidisciplinary teams is essential for optimized nutrition care. Multidisciplinary teams can collaboratively establish nutrition phenotype priorities, particularly when competing phenotypes are present, and provide tailored solutions for practical application when implementation attempts are unsuccessful.

This review has several limitations. First, the narrative review rather than systematic review study design was utilized, leading to a less formal literature review process (including the lack of bias assessment etc.), which are traditionally used for systematic reviews. Second, the nutrition phenotype framework itself was derived from author consensus informed by clinical experience and the literature reviewed, rather than through a formal Delphi process or other structured consensus and has not been validated prospectively. This should be regarded as a hypothesis-generating clinical tool rather than a validated diagnostic classification. Furthermore, the six phenotypes are not mutually exclusive, and many patients with IBD may exhibit features of more than one phenotype simultaneously. Finally, this review focused on dietary and nutritional management and did not address related domains such as the gut microbiome, psychosocial determinants of eating behavior, or health equity and food access barriers to implementation, each of which warrants dedicated future review.

Despite these limitations, the nutrition phenotyping described in this manuscript provides a practical framework for individualized, evidence-informed dietary management in IBD. By aligning interventions with immune, metabolic, anatomic, and symptomatic diversity, clinicians can better address the spectrum of nutritional needs encountered in practice. Embedding nutrition within multidisciplinary care pathways and, in the future, precision-medicine models, represents a key opportunity to enhance disease control, support patient-centered care, and advance the role of diet as a core therapeutic pillar in IBD management.

## 5 Future directions

Future studies are needed to translate the nutrition phenotype framework into routine clinical practice. Formal validation through a structured consensus process involving gastroenterologists, dietitians, and patients may help refine phenotype definitions and establish clinically meaningful diagnostic criteria. Incorporation of phenotype identification into clinical workflows, such as electronic health record–based prompts during nutrition screening or IBD clinic visits, could facilitate consistent assessment and longitudinal reassessment as patients transition between phenotypes over time. The framework may also provide a foundation for future clinical trials by enabling stratification of participants according to nutrition phenotype, thereby reducing population heterogeneity and improving evaluation of dietary interventions within more homogeneous patient groups. Such an approach may help identify which patients are most likely to benefit from specific nutritional therapies. In addition, integration of emerging precision nutrition tools, including microbiome profiling, metabolomics, and host genomics, may further refine phenotype classification and support more individualized dietary recommendations. Prospective studies evaluating whether phenotype-guided nutritional management improves clinical, nutritional, and quality-of-life outcomes compared with standard care are an important next step toward more global precision nutrition care for patients with IBD.
